# Consequences of cervical pessary for subsequent pregnancy: follow‐up of randomized clinical trial (ProTWIN)

**DOI:** 10.1002/uog.24821

**Published:** 2022-06-01

**Authors:** E. V. J. van Limburg Stirum, S. J. Zegveld, N. E. Simons, M. A. de Boer, E. Pajkrt, B. W. J. Mol, M. A. Oudijk, J. van 't Hooft

**Affiliations:** ^1^ Department of Obstetrics and Gynecology, Amsterdam UMC location University of Amsterdam Amsterdam The Netherlands; ^2^ Amsterdam Reproduction and Development Institute Amsterdam The Netherlands; ^3^ Department of Obstetrics and Gynecology, Amsterdam UMC location Vrije Universiteit Amsterdam The Netherlands; ^4^ Department of Obstetrics and Gynecology Monash University Melbourne Victoria Australia; ^5^ Aberdeen Centre for Women's Health Research, School of Medicine University of Aberdeen Aberdeen UK

**Keywords:** follow‐up, pessary, preterm birth, quality of life, twin pregnancy

## Abstract

**Objective:**

To evaluate the effect of cervical pessary, as a strategy to prevent preterm birth (PTB), on the outcome of subsequent pregnancy and maternal quality of life 4 years after the index twin pregnancy.

**Methods:**

Between 2009 and 2012, the ProTWIN trial randomized women with a multiple pregnancy to pessary use *vs* standard care for the prevention of PTB. The trial showed no benefit in unselected women with a twin pregnancy, but showed a 60% reduction in poor perinatal outcomes in favor of the pessary group in the subgroup of women with a mid‐trimester short cervix (cervical length < 38 mm). All women were invited to participate in a follow‐up study 4 years after their participation in the ProTWIN trial. In this follow‐up study, maternal quality of life was assessed using the EQ‐5D‐3L questionnaire and women were asked separate questions about subsequent pregnancies. Results were compared between women who were randomized to the pessary *vs* the control group in the ProTWIN trial by calculating relative risk (RR) and 95% CI. Subgroup analysis was performed for women with a mid‐trimester short cervix (cervical length < 38 mm).

**Results:**

Of the 813 women included in the ProTWIN trial, 408 (50.2%) participated in this follow‐up study, comprising 228 randomized to the pessary group and 180 to the control group in the original trial. The median interval between participation in the ProTWIN trial and participation in this follow‐up study was 4.1 (interquartile range (IQR), 3.9–7.1) years. Ninety‐eight (24.0%) participants tried to conceive after their participation in the ProTWIN trial. Of those, 22 (22.4%) women did not have a subsequent pregnancy (no difference between pessary and control groups), seven (7.1%) women had at least one miscarriage but no live birth, and 67 (68.4%) women had at least one live birth (35 in the pessary *vs* 32 in the control group; RR, 0.93 (95% CI, 0.8–1.07)). In two women, the pregnancy outcome was unknown. Preterm delivery (< 37 weeks of gestation) of the first live birth occurred in three women in the pessary *vs* one woman in the control group (all singleton; RR, 2.57 (95% CI, 0.28–23.44)). No differences were found between the pessary and control groups in the subgroup of women with mid‐trimester short cervix, but the numbers analyzed were small. The median health state index score was 0.95 (IQR, 0.82–0.95), with no difference between the pessary and control groups.

**Conclusion:**

Our findings suggest that there are no long‐term effects of pessary use on the outcome of subsequent pregnancies and maternal quality of life. Data on obstetric outcome were limited due to the small numbers. © 2021 The Authors. *Ultrasound in Obstetrics & Gynecology* published by John Wiley & Sons Ltd on behalf of International Society of Ultrasound in Obstetrics and Gynecology.

**CONTRIBUTION**

**What are the novel findings of this work?**

This follow‐up study is the first to evaluate the effect on subsequent pregnancy of pessary use to prevent preterm birth in a multiple pregnancy within the setting of a randomized controlled trial. We found no long‐term effects of pessary use on the outcome of subsequent pregnancy and maternal quality of life 4 years after the index pregnancy.

**What are the clinical implications of this work?**

The findings of this study improve our understanding of the long‐term maternal consequences of the use of a pessary and can help better inform clinicians and pregnant women.

## INTRODUCTION

Delivery before 37 weeks of gestation, defined as preterm birth (PTB), is the most important cause of neonatal morbidity and mortality^1^. In multiple gestation, the occurrence of PTB^2^ may be as high as 53%. The prevention of PTB in this high‐risk population is therefore an important topic in obstetric medical research.

For women with a multiple pregnancy, studies show conflicting results regarding the benefit of interventions such as cerclage, progesterone and pessary^3^, ^4^, ^5^, ^6^. The ProTWIN trial^5^ assessed the effect of a cervical pessary to prevent PTB in women with a multiple gestation and showed no benefit in unselected women. However, in women with a short mid‐trimester cervix (< 38 mm) pessary use reduced the PTB rate (relative risk, 0.49 (95% CI, 0.24–0.97)) and improved neonatal outcome^5^. Although the exact working mechanism of a cervical pessary is unknown, multiple hypotheses have been postulated. The pessary may induce a shift in weight distribution of the uterus by changing the position of the cervix and preventing the cervix from further shortening and opening. Alternatively, the pessary may facilitate preservation of the mucus plug, an important barrier for ascending infections^7^, ^8^, ^9^.

The potential benefit of an intervention to prevent PTB can be evaluated directly after the intervention or in the long term, and from the child or the maternal perspective. Even though follow‐up results of children who were exposed to a pessary *in utero* during twin pregnancy have been published^10^, ^11^, information is lacking on the long‐term effects on maternal outcome. Evidence suggests that a twin pregnancy and PTB can have an impact on maternal quality of life (i.e. higher rates of depression, anxiety, parenting stress)^12^, ^13^, ^14^, ^15^, ^16^. Moreover, occurrence of PTB in a previous pregnancy can influence the outcome of a subsequent pregnancy since the risk for a subsequent spontaneous singleton PTB is increased after a twin PTB (odds ratio, 4.3–6.7), compared with a twin pregnancy delivered at term^17^, ^18^. We hypothesized that if PTB is prevented (for example by the use of a pessary), the outcome of a subsequent pregnancy might improve as well. In this follow‐up study we aimed to evaluate the effect of pessary use on maternal quality of life and the outcome of subsequent pregnancies 4 years after the ProTWIN trial.

## METHODS

This was a follow‐up study of the ProTWIN trial (NTR1858), a multicenter randomized controlled trial assessing the effect of a pessary on perinatal outcome^5^. In that study, asymptomatic women with a multiple pregnancy were randomized to therapy with pessary (*n* = 403) or standard care (*n* = 410). An Arabin pessary was placed between 16 and 20 weeks of gestation and cervical length was measured at baseline. Details and results of this multicenter study have been described elsewhere^5^. We report this follow‐up study with consideration of the STROBE checklist version 4.

### Follow‐up assessment

All women who participated in the ProTWIN trial were eligible for follow‐up. Research nurses in the participating centers were asked to check medical records for the possible occurrence of death of participating women and/or the children. Women that had lost one or two children were approached with extra care by the investigation team. Follow‐up assessment was conducted by a tertiary academic medical center in The Netherlands between June 2014 and July 2019. After consent was obtained, two questionnaires (the standardized EuroQol‐5 Dimension (EQ‐5D‐3L; https://euroqol.org/eq‐5d‐instruments/eq‐5d‐3l‐available‐modes‐of‐administration/self‐complete‐on‐digital/) survey and a questionnaire asking about subsequent pregnancies) were sent as a paper version 4 years after the delivery. Assessors were blinded to the treatment allocation of women in the ProTWIN trial. A second attempt to find the contact data of women who were not reached 4 years after the delivery was made 7 years after the index delivery. These women were interviewed via phone by two investigators (S.J.Z., J.v.H.) using the same two questionnaires. This follow‐up research study was approved by the Medical Ethics Committee of Amsterdam UMC, The Netherlands, location AMC (NL46768.018.13).

### Subsequent pregnancies

Women were asked if they desired a pregnancy following the ProTWIN trial. Information about subsequent pregnancies, method of conception (spontaneous or artificial reproductive techniques), hospital admissions because of (threatened) PTB and gestational age at delivery (including miscarriage) were asked. PTB was defined as delivery before 37 completed weeks of gestation.

### Quality of life

To assess quality of life, women were asked about their general health using the EQ‐5D‐3L survey. The questionnaire included questions related to five dimensions: mobility, self‐care, daily activities, pain symptoms and anxiety or depression. Each dimension comprised three levels of health status: Level 1, no problems; Level 2, moderate problems; Level 3, severe problems. Participants had to choose the level that best specified their present health status, resulting in a five‐number digit code. This five number digit code was subsequently converted to an index value according to the reference of the Dutch population^19^. An index score of 0 refers to a health status equivalent to death and an index score of 1 to perfect health. Additionally, women were asked to rank their perceived health status on a visual analog scale (VAS), rating from 0 (the worst thinkable health status) to 100 (the best thinkable health status)^20^, ^21^, ^22^.

### Statistical analysis

Differences in baseline characteristics of women participating in the ProTWIN follow‐up study and randomized to pessary *vs* standard care were compared using Student's *t*‐test, Mann–Whitney *U*‐test, chi‐square test or Fisher's exact test, as appropriate. The same comparisons were made for women participating in the follow‐up study and those lost to follow‐up to assess potential attrition bias.

Follow‐up outcomes, i.e. subsequent pregnancy, the five different dimensions of the EQ‐5D‐3L and VAS scores, were evaluated using descriptive statistics and comparing pessary *vs* usual care using Student's *t*‐test, Mann–Whitney *U*‐test, chi‐square test or Fisher's exact test, as appropriate. Potential confounders were visualized through a direct acyclic graph (Figure S1). The graph demonstrated no potential confounders and therefore no corrections were applied. All analyses were performed according to the intention‐to‐treat principle. Imputation of missing data was performed for 13 cases in which the exact date of interview (at 7 years) was missing. We imputed the median follow‐up date of all women approached at 7 years follow‐up.

Subgroup analysis of women with a short cervix (< 38 mm) was prespecified because in this subgroup women with a pessary had a significantly lower PTB rate in the ProTWIN trial. Also, a *post‐hoc* subgroup analysis of women with a high risk of PTB (i.e. previous PTB or neonatal death) was performed, to explore any potential long‐term benefit or harm of pessary use for subsequent pregnancies in this group. Statistical analysis was performed using IBM SPSS Statistics®, version 26 (IBM Corp., Armonk, NY, USA). A two‐sided *P*‐value of < 0.05 was considered statistically significant.

## RESULTS

**Figure 1:**
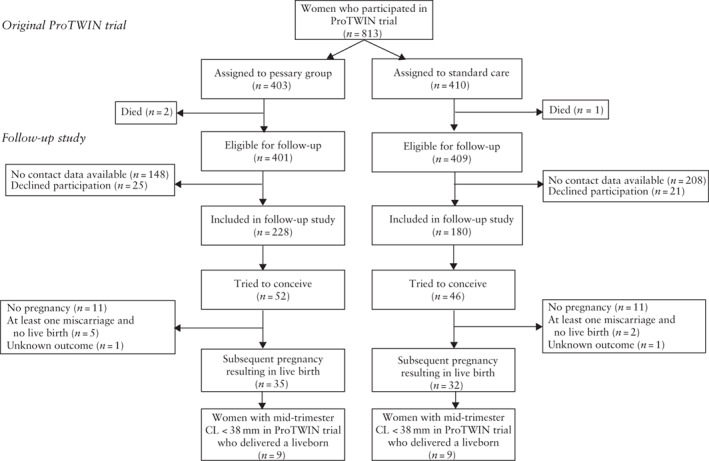
Flowchart summarizing women included in the ProTWIN trial who participated in this follow‐up study, and their pregnancy outcome. CL, cervical length.

In the original ProTWIN trial, a total of 813 women were assigned randomly to receive a pessary (*n* = 403) or standard care (control group; *n* = 410). One woman in the pessary group died during participation in the original ProTWIN trial because of sepsis (after premature rupture of membranes during placement of a cerclage)^5^. Two further women (one in the control and one in the pessary group) died from an unknown cause between randomization and the follow‐up period. A total of 356 (43.8%) women were lost to follow‐up due to missing contact data or because they could not be reached after several reminders. Another 46 (5.7%) women declined participation. In total, 408 (50.2%) women participated in this follow‐up study, comprising 228 randomized to the pessary group and 180 to the control group in the original trial (Figure 1).

Median interval from participation in the ProTWIN trial to participation in this follow‐up study was 4.1 (interquartile range (IQR), 3.9–7.1) years, with 253 women participating around 4 years after the index delivery (pessary (*n* = 136) *vs* controls (*n* = 117)) and 155 additional women participating around 7 years after the index delivery (pessary (*n* = 92) *vs* controls (*n* = 63)). There was a similar distribution in terms of follow‐up interval between the pessary and control groups (Figure S2). The median maternal age at follow‐up was 38.0 (IQR, 35.0–42.0) years in the pessary group and 38.0 (IQR, 35.0–42.0) years in the control group (*P* = 0.68).

Baseline characteristics of follow‐up participants were comparable between the pessary and control groups (Table 1). When comparing the baseline characteristics of women who participated in the follow‐up study with those of women who were lost to follow‐up, we found no difference in maternal age at randomization in the ProTWIN trial, nulliparity, smoking status, body mass index (BMI), previous fertility treatment and rate of previous PTB. However, women in the follow‐up group, compared to those lost to follow‐up, were more likely to be of European origin (92.9% *vs* 86.9%) and have higher education (66.4% *vs* 61.4%). In addition, fewer women participated in the follow‐up study after a previous PTB (55.2% in follow‐up group *vs* 61.8% in lost‐to‐follow‐up group) or after losing one or two children during the ProTWIN trial (1.7% in follow‐up group *vs* 6.7% in lost‐to‐follow‐up group) (Table 1).

**Table 1:** Baseline maternal characteristics at time of inclusion in ProTWIN trial and outcomes of original ProTWIN trial, in women who participated in this follow‐up study *vs* those lost to follow‐up and, of the 408 women included in this study, in those randomized to receive cervical pessary *vs* those who had standard care (control) in original trial

Characteristic/outcome in ProTWIN trial	Follow‐up study (*n* = 408)			ProTWIN trial (*n* = 813)		
	Pessary (*n* = 228)	Control (*n* = 180)	*P*	With follow‐up (*n* = 408)	Lost to follow‐up (*n* = 405)	*P*
Maternal age at randomization (years)	32.0 (29.0–35.8)	32.5 (30.0–36.0)	0.54	32.0 (29.0–36.0)	32.0 (29.0–35.0)	0.92
Nulliparous	129 (56.6)	102 (56.7)	0.99	231/408 (56.6)	216/404 (53.5)	0.37
Smoking during pregnancy	8/227 (3.5)	11/174 (6.3)	0.19	19/401 (4.7)	22/390 (5.6)	0.57
BMI (kg/m^2^)*	23.9 (21.5–26.8)	23.0 (21.0–25.6)	0.06	23.5 (21.3–26.3)	22.9 (21.0–26.0)	0.15
Maternal education†			0.13			0.02
High	132/189 (69.8)	97/156 (62.2)		229/345 (66.4)	135/220 (61.4)	
Middle	49/189 (25.9)	52/156 (33.3)		101/345 (29.3)	61/220 (27.7)	
Low	8/189 (4.2)	7/156 (4.5)		15/345 (4.3)	24/220 (10.9)	
European ethnic origin	209/222 (94.1)	159/174 (91.4)	0.29	368/396 (92.9)	331/381 (86.9)	0.01
Previous fertility treatment	88/227 (38.8)	64/179 (35.8)	0.53	152/406 (37.4)	139/401 (34.7)	0.41
Triplet pregnancy	6 (2.6)	3 (1.7)	0.74	9 (2.2)	9 (2.2)	0.99
Cervical length < 38 mm	54 (23.7)	29 (16.1)	0.06	83/408 (20.3)	49/214 (22.9)	0.46
PPROM	25/197 (12.7)	17/152 (11.2)	0.67	42/349 (12.0)	27/344 (7.9)	0.07
At least one neonatal or child death before discharge	4 (1.8)	3 (1.7)	1.00	7/408 (1.7)	27/401 (6.7)	< 0.0001
Preterm birth (< 37 weeks)‡	120/226 (53.1)	104 (57.8)	0.35	224/406 (55.2)	248/401 (61.8)	0.05

Data are given as median (interquartile range), *n* (%), or *n*/*N* (%) in the case of missing data.

* Body mass index (BMI) data were available in: 371 women in the follow‐up group (208 in the pessary group and 163 in the control group) and 374 women in the group lost to follow‐up.

† Maternal education (high *vs* middle and low education level): low level, < 5 total years postelementary schooling; middle level, 5–8 total years postelementary schooling; high level, > 8 total years postelementary schooling.

‡ Women who experienced a preterm birth before the ProTWIN pregnancy and/or delivered prematurely in the pregnancy of the ProTWIN trial.

PPROM, preterm prelabor rupture of membranes.

### Subsequent pregnancy

Ninety‐eight women (24.0%) tried to conceive after participating in the ProTWIN trial (Table 2). Of these, 22 (22.4%) women did not have a subsequent pregnancy, 7 (7.1%) women had at least one miscarriage but no live birth, and 67 (68.4%) women had at least one live birth (35 (85.4%) in the pessary group *vs* 32 (91.4%) in the control group; RR, 0.93 (95% CI, 0.81–1.07)). Two women who reported a subsequent pregnancy did not report the outcome (miscarriage or live birth). Median gestational age at the first subsequent live birth was 40 (IQR, 39.0–40.4) weeks. Three women in the pessary group delivered preterm (< 37 weeks) *vs* one woman in the control group (RR, 2.57 (95% CI, 0.28–23.44)). All women who had more than one live birth had a singleton pregnancy. No difference was found in use of artificial reproductive technologies, hospital admission for threatened PTB, maternal age at first subsequent pregnancy and interval from the ProTWIN trial to the first subsequent pregnancy between the pessary and control groups (Table 3).

**Table 2:** Pregnancy outcome of women included in this follow‐up study who tried to conceive after participating in the ProTWIN trial, according to whether they were randomized to cervical pessary or standard care (control) in the original trial

Outcome	Pessary (*n* = 228)	Control (*n* = 180)	RR (95% CI)	*P*
Women who tried to conceive after ProTWIN trial*	52/227 (22.9)	46/179 (25.7)	0.89 (0.63–1.26)	0.51
No pregnancy	11/52 (21.2)	11/46 (23.9)	0.89 (0.42–1.85)	0.74
Subsequent pregnancy	41/52 (78.8)	35/46 (76.1)	1.04 (0.84–1.28)	0.74
Miscarriage(s)†	5/41 (12.2)	2/35 (5.7)	2.13 (0.44–10.26)	0.44
Live birth(s)	35/41 (85.4)	32/35 (91.4)	0.93 (0.81–1.07)	0.44
Unknown outcome	1/41 (2.4)	1/35 (2.9)	—	—

Data are given as *n*/*N* (%), unless stated otherwise.

* Women who reported pregnancy desire or had miscarriage(s) or live birth(s) after the ProTWIN trial.

Of women with a live birth, six (pessary group (*n* = 4); control group (*n* = 2)) had a miscarriage prior to their live birth and 10 women (pessary group (*n* = 9); control group (*n* = 1)) reported desire for pregnancy even though they already had a live birth after the ProTWIN trial.

† Women with at least one miscarriage but no live birth.

RR, relative risk.

**Table 3:** Characteristics of first pregnancy after the ProTWIN trial resulting in a live birth, in 35 women randomized to receive cervical pessary and 32 randomized to standard care (control) in the original trial

Characteristic	Pessary (*n* = 35)	Control (*n* = 32)	RR (95% CI)	*P*
Maternal age (years)*	32.5 (30.8–36.0)	35.0 (32.0–37.8)	—	0.23
Interval from ProTWIN trial to pregnancy (months)†	34.0 (26.5–44.8)	33.5 (24.3–44.8)	—	0.90
Use of ART	5/32 (15.6)	9/27 (33.3)	0.47 (0.18–1.23)	0.11
Hospital admission for threatened PTB	2/26 (7.7)	2/27 (7.4)	0.98 (0.35–2.72)	1.00
Gestational age at delivery	—	—	2.57 (0.28–23.44)‡	0.62
< 28 weeks	0 (0)	0 (0)		
≥ 28 to < 32 weeks	1/35 (2.9)	0 (0)		
≥ 32 to < 37 weeks	2/35 (5.7)	1/30 (3.3)		
≥ 37 weeks	32/35 (91.4)	29/30 (96.7)		

Data are given as median (interquartile range) or *n*/*N* (%), unless stated otherwise.

* Data available in 30 women in the pessary group.

† Data available in 30 women in the pessary group and 28 women in the control group.

‡ Delivery < 37 weeks of gestation in pessary *vs* control group.

ART, artificial reproductive technique; PTB, preterm birth; RR, relative risk.

Of the 67 women with a subsequent live birth, 42 (62.7%) women had a history of PTB (e.g. preterm delivery before or during their ProTWIN pregnancy). Recurrent PTB after preterm twin pregnancy occurred in three of these 42 women (7.1%). PTB after a term twin pregnancy occurred in one of 25 women (4.0%).

### Subgroup analysis of women with a short cervix

Of the 83 women with a short cervical length (< 38 mm) in the ProTWIN trial, 25 (30.1%) tried to conceive, of whom 21 achieved a pregnancy (12 in the pessary and nine in the control group). Of these, three women (all in the pessary group) had at least one miscarriage but no live birth, and 18 had at least one live birth (9/12 (75%) in the pessary group *vs* 9/9 (100%) in the control group (RR, 0.75 (95% CI, 0.54–1.04))). Two women (22%) in the pessary group delivered preterm *vs* none in the control group (RR, 0.78 (95% CI, 0.55–1.10)) (Table S1).

### Subgroup analysis of women with other risk factors

In total, 224/406 women had a history of PTB or neonatal death (i.e. including preterm delivery or neonatal death in their ProTWIN pregnancy), comprising 120 (53.6%) women in the pessary group and 104 (46.4%) in the control group. Information was incomplete for two women. In the subgroup, 56 (25.0%) women tried to conceive, and of those who achieved a pregnancy (*n* = 47), three women had at least one miscarriage but no live birth and 42 women had at least one live birth. There were no differences between the pessary and control groups (Table S2). Three women (3/22; 13.6%) in the pessary group delivered preterm *vs* none in the control group (RR, 0.86 (95% CI, 0.73–1.02)). All women who delivered extremely preterm (< 32 weeks) in the original ProTWIN trial (5 (100%) in the pessary group *vs* 4 (100%) in the control group) delivered at term in their first subsequent live birth.

### Quality of life

The median health state index score was 0.95 (IQR, 0.82–0.95) in the pessary group and 0.95 (IQR, 0.82–0.95) in the control group. The responses of women with respect to the five health dimensions in the EQ‐5D‐3L are shown in Table 4. The median VAS score for women's perceived health status was 80.0 (IQR, 75.0–86.0) in the pessary group versus 80.0 (IQR, 75.0–90.0) in the control group. The measured health state index and VAS scores were similarly distributed between women who were followed up at 4 years and those followed up at 7 years (Figure S3).

**Table 4:** Responses with respect to the five dimensions of the EQ‐5D‐3L survey of 228 women randomized to cervical pessary and 180 randomized to standard care (control) in the ProTWIN trial who participated in this follow‐up study

Level	Mobility		Self care		Usual activities		Pain/discomfort		Anxiety/depression	
	Pessary	Control	Pessary	Control	Pessary	Control	Pessary	Control	Pessary	Control
Level 1 (no problems)	214/227 (94.3)	168/180 (93.3)	225/227 (99.1)	178/180 (98.9)	211/227 (93.0)	158/179 (88.3)	172/225 (76.4)	142/179 (79.3)	207/225 (92.0)	162/180 (90.0)
Level 2 (moderate problems)	13/227 (5.7)	12/180 (6.7)	2/227 (0.9)	2/180 (1.1)	16/227 (7.0)	21/179 (11.7)	51/225 (22.7)	31/179 (17.3)	15/225 (6.7)	17/180 (9.4)
Level 3 (severe problems)	0	0	0	0	0	0	2/225 (0.9)	6/179 (3.4)	3/225 (1.3)	1/180 (0.6)
Data missing	1	0	1	0	1	1	3	1	3	0

Data are given as *n*/*N* (%) or *n*.

## DISCUSSION

We did not find any evidence that the use of a pessary to prevent PTB in a twin pregnancy affects the outcome of subsequent pregnancies. The quality of life of women 4 years after their twin pregnancy did not differ between women who received a pessary *vs* those who did not.

This is the first follow‐up study evaluating the effect of a pessary on subsequent pregnancies (review of the literature in January 2021: PubMed search ‘pessaries’ [MeSH Terms] OR pessary [Text Word] AND (subsequent OR pregnancy) resulted in 0 hits) within the setting of a randomized controlled trial. This research question is of the outmost importance, as the evaluation of obstetric interventions often stops after the index pregnancy and data on potential long‐term effects for subsequent pregnancies are missing. Thanks to a response rate of 50%, we also managed to have a reasonable number of participants to test our main hypothesis.

This study has several limitations. First, attrition bias is a common problem of follow‐up studies that also likely applies to this study. We received questionnaires from more women with a higher education and of European origin, as compared to the group lost to follow‐up. This attrition bias could have caused an overestimation of women's quality of life and underrated subsequent PTB due to the fact that maternal education and ethnicity are associated with risk for PTB and quality of life^23^. The lower participation number of women who delivered preterm (or experienced neonatal death) in the ProTWIN trial also likely impacted the subsequent PTB rate and quality of life found in this study^18^, ^24^. Attrition bias hampers the generalizability of the results. It is also important to note that the measured quality of life is probably mediated by the outcomes of the index pregnancy (i.e. PTB or other short/long‐term problems). It is therefore too simplistic to say that the quality of life measured in this study relates to pessary use itself, but is more related to pessary use and its potential consequences on pregnancy outcome. Second, the variance in timing of follow‐up could have resulted in underestimation of the total number of subsequent pregnancies in this follow‐up sample. However, we do not think this will have had major impact on our final conclusions, as the main analysis focusses on the first subsequent pregnancy, most likely happening within 4 years after the twin pregnancy^17^ and proportion of women with a live birth was equally distributed between 4‐ and 7‐years' follow‐up. By extending our follow‐up period, we managed to extend our sample size, and therefore the follow‐up rate. Third, in this follow‐up study, women were not asked about their medical history and present health status (e.g. weight gain or cervical conization between participation in the original trial and follow‐up), which could affect gestational age at birth of a subsequent pregnancy as well as quality of life. Therefore, it is unknown if women received obstetric interventions (e.g. progesterone, cervical cerclage or a pessary) in the subsequent pregnancy, which could also influence gestational age at birth. Moreover, cervical necrosis and cervical laceration are described in the literature as complications of pessary use in pregnancy^25^, ^26^. When this occurs, the risk for PTB in a subsequent pregnancy may be high. In this study, information about this complication is lacking and, therefore, the effect of this complication on the outcome of subsequent pregnancies could not be assessed.

The rate of recurrent PTB of a singleton gestation after a preterm twin pregnancy is reported to be 7.3–19.8% in the literature^17^. This is higher than the recurrent PTB rate of 7.1% measured in our population. The reason for this difference may be that the proportion of women who delivered preterm in the ProTWIN trial was higher in the lost‐to‐follow‐up group. The risk of PTB of a subsequent singleton pregnancy after a term twin gestation is between 0.8–6.9% in the literature^17^, which is comparable to the results in our population (4.0%).

Our results suggest a lower self‐rated health 4 years after a twin pregnancy compared to the Dutch reference population of women aged 35–49 years, as reflected by a VAS score of 80.0 (IQR, 75–86) in our population *vs* 89.1 (95% CI, 87.0–91.3) in the Dutch reference population. Interestingly, the quality of life scoring according to the five health dimensions was comparable with the general Dutch population (median health state index of 0.95 (IQR, 0.82–0.95) in our population compared to a mean of 0.92 (95% CI, 0.90–0.94) for women between 35–49 years of age in the general Dutch population)^23^. This is remarkable, considering that a review by Wenze *et al*.^14^ showed worse mental health outcome (i.e. higher rates of depression, anxiety, parenting stress) in parents of multiples compared to parents of singletons in the postpartum period and up to 5 years after childbirth. We found no other studies on twins measuring quality of life using the EQ‐5D (literature search May 2021 using searching terms ‘quality of life [MeSH Terms] or Maternal Health [MeSH Terms]) and (eq‐5d* or (Surveys and Questionnaires [MeSH Terms]) or EuroQol*) and (twin [MeSH Terms] or (multiple and (gestation or pregnancy)’). Therefore, it is difficult to say if the difference in this outcome in this follow‐up study can be explained by the aforementioned attrition bias, or because of the use of a different questionnaire.

In conclusion, in this follow‐up study of women randomized to pessary or standard care during multiple pregnancy, we found no evidence that the use of a cervical pessary affects the outcome of subsequent pregnancies, although data on obstetric outcomes (e.g. PTB) were limited due to the small numbers. Quality of life in our sample was comparable to the reference population in The Netherlands and did not differ between women who used a pessary *vs* those who did not. It would be valuable to investigate this research question in other treatment strategies that aim to prevent PTB, such as cerclage or progesterone.

## Disclosures

B.W.M. is supported by a NHMRC Investigator grant (GNT1176437). B.W.M. reports consultancy for ObsEva and has received research funding from Guerbet, Ferring and Merck. The original ProTWIN trial was funded by ZonMW grant 200310004. We did not receive any funding for this follow‐up research.

## Supporting information


**Figure S1** Direct acyclic graph.
**Figure S2** Distribution of interval from participation in the ProTWIN trial to inclusion in this follow‐up study for the pessary and control groups.
**Figure S3** Distribution of the visual analog scale and health state index scores in respect to follow‐up (FU) interval from the ProTWIN trial.
**Tables S1 and S2** Pregnancy outcome of women who tried to conceive after the ProTWIN trial and characteristics of live births, in women with cervical length < 38 mm in the original trial (Table S1) and in those with risk factors i.e. history of preterm birth or neonatal death during the ProTWIN trial (Table S2)

## Data Availability

Data of this study are available from the corresponding author upon reasonable request.
