# Supplementary material for: Consequences of cervical pessary for subsequent pregnancy: follow‐up of randomized clinical trial (ProTWIN)
Source: Ultrasound Obstet Gynecol. 2022 Jun 1;59(6):771–7. doi: 10.1002/uog.24821 (PMC9328140; doi:10.1002/uog.24821)
Supplement: Supplementary file 1 — Figure S1 Direct acyclic graph. Figure S2 Distribution of interval from participation in the ProTWIN trial to inclusion in this follow‐up study for the pessary and control groups. Figure S3 Distribution of the visual analog scale and health state index scores in respect to follow‐up (FU) interval from the ProTWIN trial. Tables S1 and S2 Pregnancy outcome of women who tried to conceive after the ProTWIN trial and characteristics of live births, in women with cervical length < 38 mm in the original trial (Table S1) and in those with risk factors i.e. history of preterm birth or neonatal death during the ProTWIN trial (Table S2) [file UOG-59-771-s001.docx]

**Figure S1** Direct acyclic graph


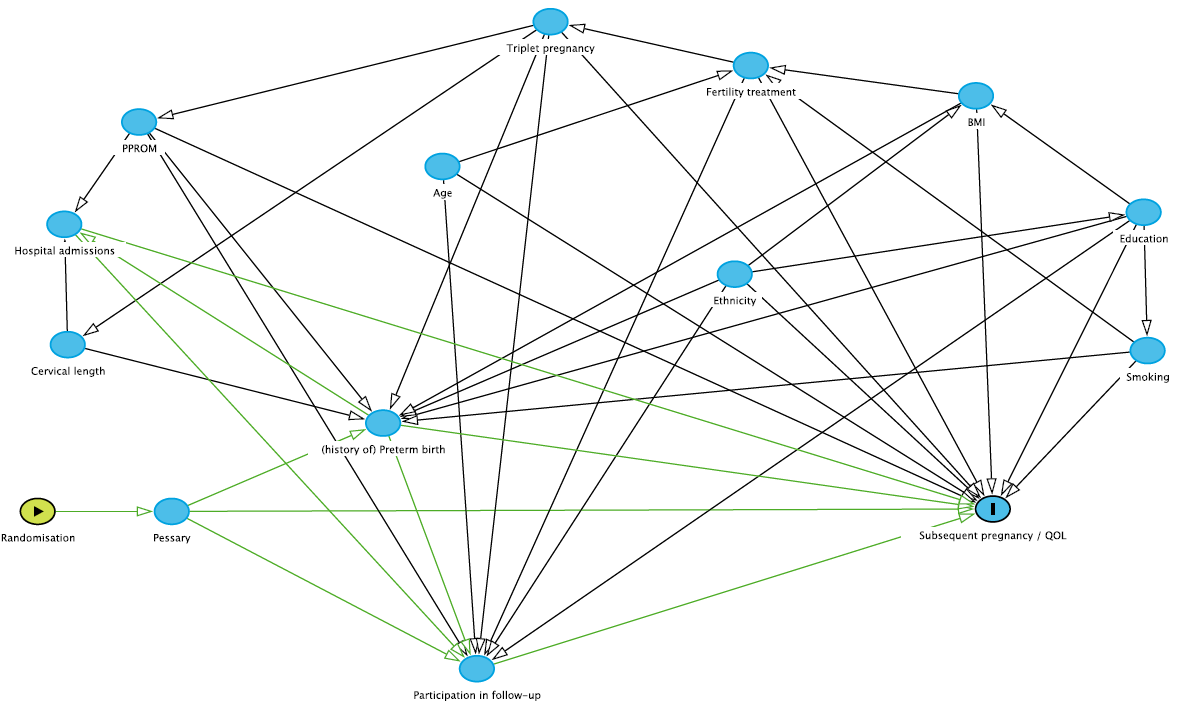


**Figure S2** Distribution of interval from participation in the ProTWIN trial to inclusion in this follow-up study for the pessary and control groups.


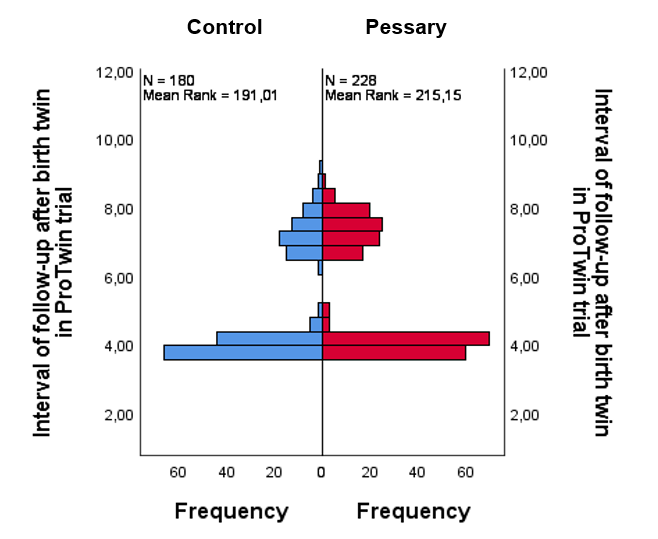


**Figure S3** Distribution of the visual analog scale and health state index scores in respect to follow-up interval from the ProTWIN trial. FU, follow-up.


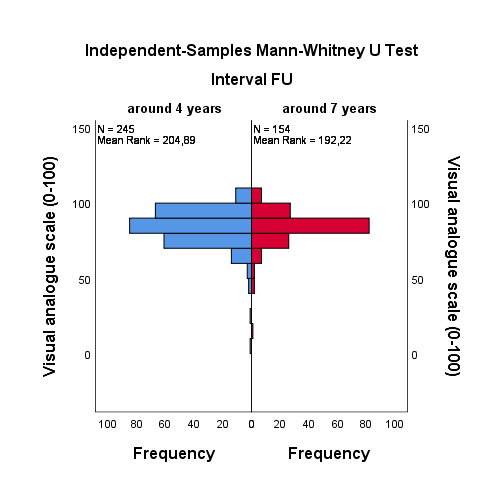


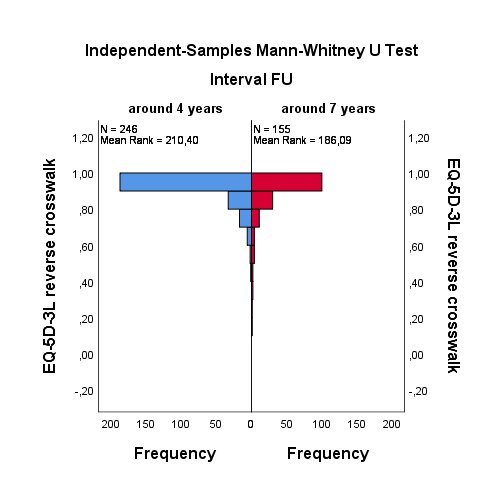


**Table S1** Pregnancy outcome of women who tried to conceive after the ProTWIN trial and characteristics of live births in women with cervical length < 38 mm in the original trial

| **Outcomes in follow-up group with cervical length <38mm** | **n/n^*^** | **Pessary  n=54** | **Control n=29** | **RR (95% CI)** | **P-value** |
| --- | --- | --- | --- | --- | --- |
| Women who tried to conceive after ProTWIN, n(%)^†^  No pregnancy  Subsequent pregnancy   - Miscarriage(s)^‡^  - Live birth(s) | 54/28  15/10  15/10 12/9 12/9 | 15 (27.8)  3 (20.0)  12 (80.0) 3 (25.0) 9 (75.0) | 10 (35.7)  1 (10.0)  9 (90.0) 0  9 (100) | 0.78 (0.40-1.50)  2.00 (0.24-16.61)  0.89 (0.64-1.23) 0.75 (0.54-1.04) 0.75 (0.54-1.04) | 0.46  0.63  0.63 0.23 0.23 |
| **Characteristics of subsequent pregnancy resulting in first live birth after ProTWIN** | **n/n^*^** | **Pessary  n=9** | **Control n=9** | **RR (95% CI)** | **P-value** |
| Maternal age, median (IQR) | 6/8 | 30.5 (29.3-32.0) | 34.5 (29.8-36.5) |  | 0.09 |
| Interval of subsequent pregnancy after ProTWIN trial in months, median (IQR) | 6/8 | 26.0 (22.5-35.3) | 30.5 (20.0-47.8) |  | 0.65 |
| Use of ART^§^, n(%) | 9/8 | 0 | 3 (37.5) | 1.60 (0.94-2.74) | 0.08 |
| Hospital admission for threatened preterm birth, n(%) | 4/8 | 1 (25.0) | 1 (12.5) | 0.60 (0.11-3.21) | 1.00 |
| Gestational age at delivery in weeks, n(%)^¶^  <28 wk  <32 wk  <37 wk  ≥ 37 wk | 9/9 | 0 (0.0) 1 (11.1) 1 (11.1) 7 (77.8) | 0 (0.0) 0 (0.0) 0 (0.0) 9 (100.0) | 0.78 (0.55-1.10) | 0.47 |

*number of analyzed mothers without missing data. Pessary group / control group.
†women who reported pregnancy desire or had miscarriages or had a live birth after the ProTWIN trial.
‡miscarriage presented as the number of women with at least one miscarriage but no live births.
§artificial reproductive therapy.
¶weeks of gestation during delivery: delivery ≥37 weeks of gestation.

**Table S2** Pregnancy outcome of women who tried to conceive after the ProTWIN trial and characteristics of live births, in women with risk factors i.e. history of preterm birth or neonatal death during the ProTWIN trial

| **Outcomes in follow-up group with history of premature birth or neonatal death*** | **n/n^**^** | **Pessary  n=120** | **Control n=104** | **RR (95% CI)** | **P-value** |
| --- | --- | --- | --- | --- | --- |
| Women who tried to conceive after ProTWIN, n(%)^†^  No pregnancy  Subsequent pregnancy   - Miscarriage(s)^‡^  - Live birth(s)  - Unknown outcome | 119/ 103  28/28  28/28 25/22 25/22 25/22 | 28 (23.5)  3 (10.7)  25 (89.3) 2 (8.0) 22 (88.0) 1 (4.0) | 28 (27.2)  6 (21.4)  22 (78.6) 1 (4.5) 20 (90.9) 1 (4.5) | 0.87 (0.55-1.36)  0.50 (0.14-1.80)  1.14 (0.90-1.43) 1.75 (0.17-17.95) 0.96 (0.83-1.12) | 0.53  0.47  0.47 1.00 1.00 |
| **Characteristics of subsequent pregnancy resulting in first live birth after ProTWIN** | **n/n^**^** | **Pessary  n=22** | **Control n=20** | **RR (95% CI)** | **P-value** |
| Maternal age, median (IQR) | 18/20 | 32.0 (30.8-35.3) | 35.0 (31.3-38.8) |  | 0.33 |
| Interval of subsequent pregnancy after ProTWIN trial in months, median (IQR) | 18/18 | 34.5 (27.0-44.8) | 35.0 (28.3-51.0) |  | 0.99 |
| Use of ART^§^, n(%) | 22/18 | 2 (9.1) | 6 (33.3) | 0.27 (0.06-1.19) | 0.11 |
| Hospital admission for threatened preterm birth, n(%) | 15/17 | 2 (13.3) | 1 (5.9) | 0.67 (0.27-1.65) | 0.59 |
| Gestational age at delivery in weeks, n(%)^¶^  <28 wk  <32 wk  <37 wk  ≥ 37 wk | 22/19 | 0 (0.0) 1 (4.5) 2 (9.1) 19 (86.4) | 0 (0.0) 0 (0.0) 0 (0.0) 19 (100.0) | 0.86 (0.73-1.02) | 0.24 |

*All women with neonatal death in the ProTWIN trial delivered preterm.
**number of analyzed mothers without missing data. Pessary group / control group.
†women who reported pregnancy desire or had miscarriages or had a live birth after the ProTWIN trial.
‡miscarriage presented as the number of women with at least one miscarriage but no live births.
§artificial reproductive therapy.
¶weeks of gestation during delivery: delivery ≥37 weeks of gestation.
